# Incredible Combination of Lifestyle Modification and Herbal Remedies for Polycystic Ovarian Syndrome Management

**DOI:** 10.1155/2023/3705508

**Published:** 2023-06-20

**Authors:** Acharya Balkrishna, Maneesha Rana, Shalini Mishra, Deepika Srivastava, Rohit Bhardwaj, Shalini Singh, Satyendra Kumar Rajput, Vedpriya Arya

**Affiliations:** ^1^Patanjali Yogpeeth Trust, Kathmandu, Nepal; ^2^Patanjali Herbal Research Department, Patanjali Herbal Research Institute, Haridwar 249405, Uttarakhand, India; ^3^Department of Pharmaceutical Sciences, Gurukula Kangri (Deemed to be University), Haridwar, Uttarakhand 249404, India

## Abstract

A relatively frequent endocrine-metabolic illness called polycystic ovarian syndrome (PCOS) is characterized by polycystic ovaries, persistent anovulation, and hyperandrogenism, which cause symptoms such as irregular menstruation, infertility, and hirsutism. PCOS is linked to obesity, insulin resistance, and increased amounts of androgens, or male hormones. The sedentary lifestyle, dietary fluctuations, inactivity, and stress are other contributing variables. According to estimates from India in 2021, around 22.5% of women, or one in five Indian women, suffer from PCOS. Evidence-based medical care for PCOS places a strong focus on a multidisciplinary approach, as standard pharmacological treatment frequently targets a single symptom, may be contraindicated, has adverse effects, and is ineffective in certain circumstances. However, long-term treatments have drawbacks and are likely to be ineffective, making complementary and alternative therapies a worthwhile choice. Yoga science is a thorough treatment plan for a healthy body and mind that may eradicate PCOS's primary causes, stress and obesity. Some common herbal remedies, including *Foeniculum vulgare*, *Tinospora cordifolia*, *Asparagus racemosus*, *Ocimum tenuiflorum*, *Areca catechu*, and *Lepidium meyenii*, have been highly regarded sources that have the benefits of lowering PCOS as well as having hypoglycemic and antiobesity effects. In light of existing literature, women with PCOS experienced symptomatic relief, improvement in hormonal balance, and the quality of life by utilizing yoga practices as well as herbal remedies. In conclusion, combining lifestyle modifications with herbal remedies can be used in the management of PCOS as a holistic approach. Therefore, this review opens a new window for researchers all across the world to validate such findings.

## 1. Introduction

About 6–8% of women worldwide are affected by polycystic ovarian syndrome (PCOS), which was first identified in 1935 by Stein and Leventhal and is linked to polycystic ovarian morphology, chronic oligoanovulation, psychological problems, and metabolic abnormalities, particularly compensatory hyperinsulinemia and insulin resistance [1]. According to estimates from the World Health Organization (WHO), PCOS impacted 116 million women (3.4%) globally in 2012. Estimates of PCOS prevalence vary widely around the globe, from 2.2% to as high as 26% [2]. Experts estimate that 10% of women in India have PCOS, although there is currently no accurate published statistical information on the prevalence of PCOS in India [3]. In India nowadays, PCOS affects roughly 22.5% of women or one in every five Indian women [4].

**Figure 1:**
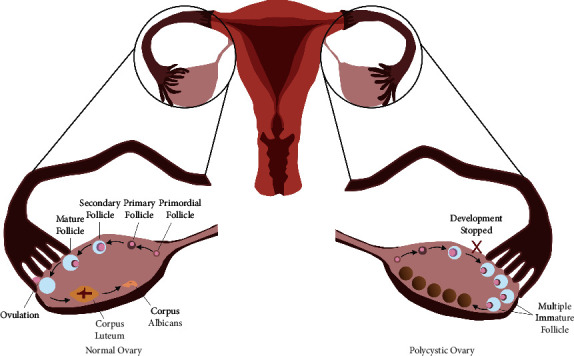
View of a normal and polycystic ovary.

Many doctors struggle to recognize this widespread condition since there are no clear diagnostic criteria [1]. Ovarian follicles normally contain egg cells, which are released during ovulation, whereas polycystic ovarian syndrome is characterized by aberrant hormone levels that hinder follicles from developing and maturing in order to produce egg cells, as depicted in Figure 1. However, reproductive abnormalities are more likely in women with PCOS [5]. This condition, also known as the Stein–Leventhal syndrome, is a significant contributor to infertility in women. Numerous theories explain the hormonal imbalance, which has no clear origin. Polycystic ovaries, hyperandrogenism, and several metabolic abnormalities (including insulin resistance and hyperinsulinemia) [6] are its defining traits. The current standard of treatment for PCOS in women includes everything from medication therapies to lifestyle changes. Diet, weight reduction, and exercise are all linked to lifestyle modifications. Modern medicines including metformin, thiazolidinediones, and estrogen-progestin combinations as well as antiandrogens such as spironolactone and flutamide (oral contraceptives) are generally used to treat PCOS [7]. Despite being successful, such therapy is expensive and may have a number of adverse effects, including gastrointestinal issues, weight gain, irregular menstruation, and elevated insulin resistance [8]. Since ancient times, medicinal plants have drawn special attention. Today, a number of studies have been conducted, leading to the discovery of valuable and advantageous medicinal plants [9]. In order to better understand the side effects of these therapies and how to identify them, a lot of research has been done on herbal remedies, including case studies, randomized controlled trials, and animal experiments. Insulin resistance is frequently present in PCOS patients, who exhibit testosterone levels that are roughly double those of typical women [10]. Several herbs including jeera powder (cumin seed powder), green tea, chia seeds, anise, fenugreek seeds, black seed oil, fennel seeds, flax seed, evening primrose oil, cinnamon powder, and turmeric have been highly regarded for their benefits in lowering PCOS as well as their antiobesity and hypoglycemic effects [11]. Integrated-pathy, *i.e*., yoga and traditional herbal remedies can show a better effect for treating polycystic ovarian syndrome, as they have since ancient times.

This review is focused on the most current information and research related to PCOS, its pathobiology, and the individual significance of herbs and lifestyle changes through yoga practices with various clinical evidence as well as the possibility of the combinational efficacy of herbal treatment and yoga therapy for better effectiveness.

## 2. Pathobiology of Polycystic Ovary Syndrome

### 2.1. Symptoms

**Figure 2:**
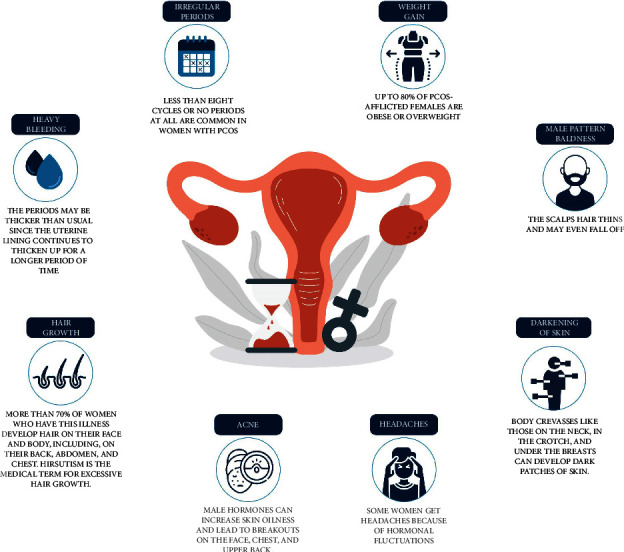
Different symptoms of polycystic ovarian syndrome.

Some women begin to experience symptoms right before the beginning of their menstruation. Others do not realize they have PCOS until they have gained a lot of weight or had trouble becoming pregnant. Although not all women with PCOS have the exact same symptoms, hormonal imbalance is a common characteristic of PCOS sufferers. For instance, if anyone has high amounts of the male hormone androgen, they will likely battle with irregular periods, facial hair, hormonal acne, and scalp hair loss [12]. Some of the most common PCOS symptoms are incorporated in Figure 2.

### 2.2. Causes

**Figure 3:**
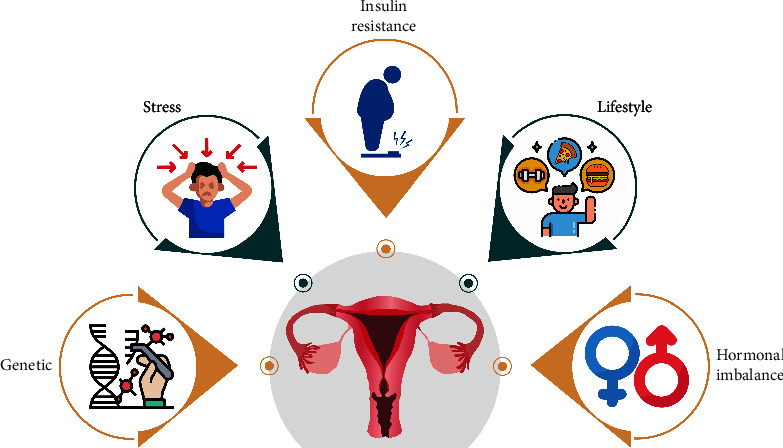
Various causes of polycystic ovarian syndrome.

Although the specific etiology of PCOS is unknown, it appears to be linked to genetics, family history, hormones that are elevated during the process of our growth in the womb before birth, and lifestyle or environmental factors [13]. The main factors responsible for the development of PCOS are mentioned in Figure 3.

Since PCOS has not yet been linked to a single gene, the connection is probably complicated and likely involves several genes. A direct female relative with PCOS, such as a mother, aunt, sister, or daughter, is 50% more likely to have it in PCOS-positive women [14]. Families with PCOS are also frequently affected by type 2 diabetes [15]. The symptoms and indications of PCOS are brought on by an imbalance of the androgens (male-type hormones like testosterone) and hormones insulin in the body [16]. One of insulin's functions in the body is to prevent excessive blood glucose (also known as sugar or energy) increases after meals [15]. It accomplishes this by “unlocking” the body's cells and enabling glucose to enter the cells from the circulation. The amount of glucose in the blood is reduced as a result. Insulin resistance affects 85% of all women with PCOS [17]. When a person has insulin resistance, the cells in their body cease reacting to insulin as they should and instead inhibit glucose absorption [18]. This indicates that your body is not successfully using the insulin that is available to maintain stable blood sugar levels. The body responds by creating extra insulin since the insulin is not functioning properly. Increased insulin levels lead to an increase in the ovaries' synthesis of androgens like testosterone [19]. Being overweight, whether due to food, physical inactivity, or both, is a lifestyle factor that contributes to insulin resistance. However, women of various weight ranges can develop insulin resistance because of hereditary reasons [16]. Approximately 95% of overweight and 75% of lean women with PCOS exhibit insulin resistance, according to the evidence [20]. It is critical to comprehend what insulin resistance is because it contributes significantly to PCOS symptoms. However, it is also critical to be aware that it may decrease.

Regular yoga sessions and a balanced diet are crucial for maintaining and lowering insulin resistance and can significantly lessen PCOS symptoms. Androgens, sometimes referred to as “man hormones,” are typically found in both women and men, but in considerably lower concentrations in the latter [21]. Androgens are produced at trace levels in the bodily tissues of all females, including the adrenal glands and the ovaries [22]. Women with PCOS have symptoms including increased body hair growth, scalp hair loss, and acne as a result of elevated androgen levels [23]. They also lead to signs like abnormal ovulation and menstrual cycles. PCOS can affect both thin and overweight women. However, obesity and being overweight are more likely among PCOS-positive women [24]. The symptoms of PCOS and insulin resistance are both made worse by excess weight, which is also believed to play a significant role in the onset of PCOS [25].

### 2.3. Histopathological Features

**Figure 4:**
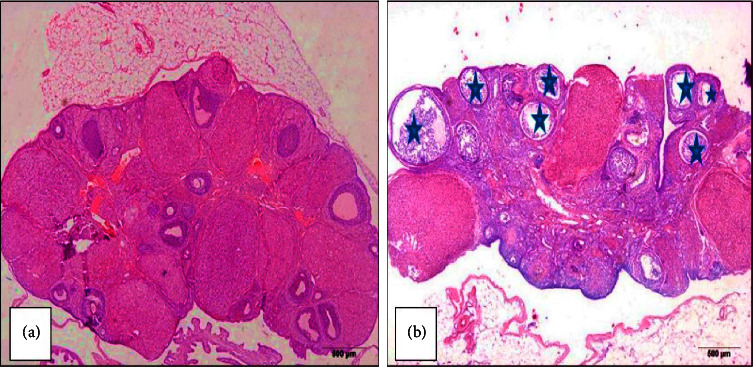
Histological slide of polycystic ovarian syndrome (PCOS): (a) normal follicles; (b) multiple cysts reproduced from Alahmadi et al. [27] under the terms and conditions of the Creative Commons attribution (CCBY) license (https://creativecommons.org/licenses/by/4.0/).

Women with PCOS experience having the following changes to their ovarian tissues [26]: an abundance of multiple, sclerotic, enlarged, and cystic follicles; whole ovarian hypertrophy; a thickened capsule >100 *μ*m; more subcapsular follicle cysts; a lack of corpora lutea or albicantia; hyperplasia and fibrosis of the ovarian stroma; and early lutein. The microscopic image of a female ovarian tissue that has PCOS such as the presence of many cysts and degenerated follicles [27], is shown in Figure 4.

### 2.4. Complications

**Figure 5:**
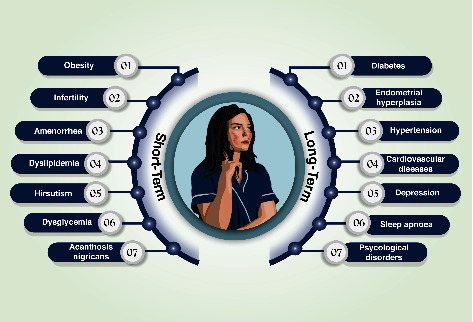
Various complications related to polycystic ovarian syndrome.

The short- and long-term impacts of PCOS on women's health are because of a higher incidence of early difficulties including worsened fertility and obstetric outcomes and an increased rate of late issues as well as elevated cardiovascular, metabolic, and cancer risks [28], as shown in Figure 5. Due to the varied character of the condition, the ambiguous nature of the pathogenetic pathways, and the existence of confounding variables, such as obesity, it is exceedingly difficult to precisely identify the degree of these difficulties. Furthermore, PCOS phenotypes in women vary over the course of a woman's life. As a result, alterations in ovarian function and metabolic control may alter how the illness manifests itself and may contribute to the morbidity of the condition during the late reproductive years and menopause [29].

### 2.5. Pathophysiology

**Figure 6:**
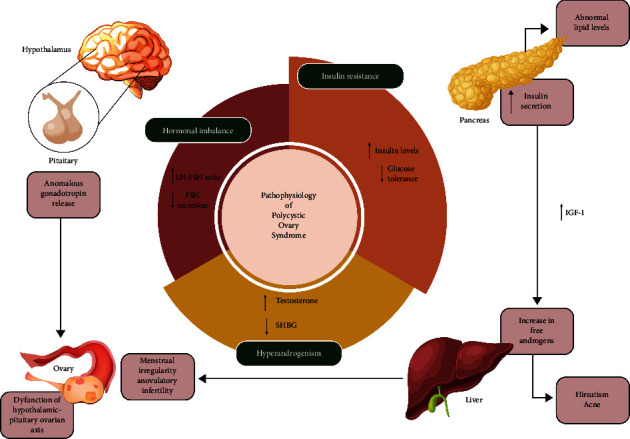
Pathogenesis of polycystic ovarian syndrome.

PCOS is primarily characterized by infertility, hyperandrogenism, lack of ovulation [30], increased levels of LH [31], increased insulin resistance, decreased sex hormone-binding globulin (SHBG) [32, 33], and hirsutism [34], which can be seen and diagnosed by ultrasonography and laboratory tests. The exact cause of PCOS has not yet been identified. Serum concentrations of androgens such as androstenedione, testosterone, and dehydroepiandrosterone are likely elevated in PCOS-afflicted women due to disruptions in the secretion rate and metabolism of androgens and estrogen [34, 35]. Additionally, it is extremely possible that some issues may arise, such as hyperinsulinemia and environmental insulin resistance. Different degrees of these problems cause obesity. The defective signaling pathway of the insulin receptor can lead to insulin resistance. As a result, the cell's ability to use insulin is compromised, which results in an additional rise in insulin release to make up for its deficiency. Adiponectin levels fall in PCOS patients with insulin resistance, which amplifies the influence of gonadotropins on ovarian function [35]. The frequency of GnRH increases as a result of insulin resistance and the excessive rise in insulin levels that follows, which raises LH/FSH levels.  Figure 6 provides a brief summary of these hormonal alterations in the theca cells and granulosa cells (GCs), which result in an increase in androgen production and a decrease in estradiol synthesis, impede follicle growth, hamper ovulation, and ultimately contribute to the development of PCOS [36].

PCOS is a hormonal condition that affects a lot of women who are fertile. Menstrual cycles in women with PCOS may be irregular or protracted, and androgen levels may be excessive. It is possible for the ovaries to grow many tiny fluid-filled sacs (follicles) but not consistently discharge eggs [37].

## 3. Search Engines

**Figure 7:**
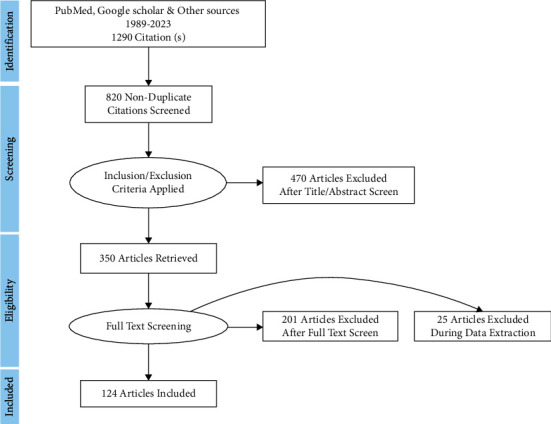
Flowchart depicting the entire review process (PRISMA).

A total of 1290 citations were discovered between the years of 1989 and 2023 after investigations into a number of search engines, including PubMed, Google Scholar, along with some other validated websites. Reviewers examined 820 abstracts for pertinent search criteria after eliminating duplicates. Additionally, 470 references were disregarded based on title and article screening, and 350 publications were retrieved based on the pathobiology of polycystic ovarian syndrome, potential treatments including herbal and yoga science, and clinical evidence. Furthermore, 201 references overall did not meet the requirements for inclusion in the full-text review since 25 articles were excluded from the data extraction procedure. In the full-text reviews, only 124 references were reviewed.  Figure 7 depicts the abovementioned search process.

## 4. Polycystic Ovary Syndrome Rectified by Lifestyle Modification

A desk job, a diet high in fried foods, processed meats, hot dogs, and sausages, as well as the consumption of too much sugar and carbonated beverages, all contribute to PCOS by causing insulin and hormonal imbalances that stimulate androgen receptors found outside the ovary. Patients with PCOS can increase their insulin sensitivity and reduce the weight by making lifestyle changes including daily yoga practices, eating a balanced diet, and limiting their intake of dairy and fast food [38]. Therefore, the primary line of treatment for PCOS patients should be lifestyle change. Unfortunately, all of these therapies only work temporarily, so maintaining this regimen throughout the course of a person's life is necessary for long-term weight loss. The outcomes are found to be unsustainable in 90 to 95% of the situations. Bariatric surgery is the sole option for treating those who are very obese and want to lose weight consistently [39]. The National Institutes of Health's most recent recommendations state that individuals who need bariatric surgery must also have additional major medical issues in addition to a body mass index of 40 or less [40]. In light of the additional symptoms of hormonal and endocrine problems that affect women with PCOS, bariatric surgery has been shown to significantly reduce PCOS symptoms and may even result in a cure [41].

Although we cannot entirely avoid stress in our daily lives, we can certainly increase our ability to handle it. Practicing yoga regularly might help you reduce stress. Even unintended weight gain in women can be caused by stress. The body stores fat as a result of cortisol. The synthesis of the stress hormone cortisol is reduced when one enters a tranquil mood. Through breathing exercises that completely calm the body, yoga-like Shavasana (Corpse pose) reduces any stress [42]. The consequences of hormone imbalance may be countered by relaxation, which can also deal with unpleasant feelings, irritation, and frequent mood swings [43]. The simplest approach to staying in shape is to perform Surya namaskar which helps in weight loss, improves the lipid profile, promotes lush hair and glowing skin, achieves a lower waist-to-hip ratio, strengthens muscles and joints, brings down blood sugar levels, and regulates the menstrual cycle [44]. Effective stress management, a decreased propensity for stress eating, and enhanced bodily awareness—particularly in relation to hunger, satiety mindfulness, and mindful eating—are some of the ways yoga may aid in the weight reduction process [45].

In turn, more muscle mass aids in the fight against insulin resistance, which is one of the cornerstones of PCOS control [46]. A vigorous yoga practice raises the heart rate, resulting in cardiovascular exercise and weight reduction. Asanas and pranayama encourage hormonal balance and profound relaxation, assisting in regulating the adrenal and cortisol levels in anxious PCOS minds and bodies [47]. We may achieve a disease-free body and a healthy mind by incorporating yoga and meditation early in life. Pranayamas (breathing exercises) are potent methods that help keep the mind calm [42]. Asanas (yoga postures) such as Badhakonasana (butterfly pose) and Suptabadhakonasana (reclined bound angle) were developed for PCOS to help open up the pelvic region and encourage relaxation which in turn helps to reduce stress and relieve discomfort related to menstruation [42]. A soothing sitting yoga position known as Bharadvajasana (Bharadvajasana twists) helps to cure PCOS symptoms by stretching the spinal column [45]. By reviving the spine, muscles, and neurological system, regular practice of this yoga posture helps to correct menstrual difficulties, regulate blood pressure, and promote calm all around [48]. Another fundamental yoga posture is Chakki Chalanasana, or the “mill churning pose,” which massages the uterus, reproductive organs, liver, kidneys, and pancreas in addition to altering the functioning of the endocrine gland to enable optimal hormone release [49]. Both yoga and a balanced diet can aid in weight loss. Along with this, practice certain calming meditations that have a profound impact on the body's physiology and aid in systemic detoxification and stress reduction [42].

In teenage females with PCOS, yoga was found to be more beneficial than traditional physical activity in improving glucose, insulin, and lipid levels, including insulin resistance values, irrespective of anthropometric changes [17]. In teenage PCOS, a comprehensive yoga program lasting 12 weeks was shown to be substantially superior to physical activity in lowering LH, AMH, and testosterone, the mFG score for hirsutism, as well as decreasing menstruation frequency with no discernible changes in body weight, prolactin, and FSH [50]. Regularly practicing mindful yoga is a beneficial supplementary therapy option for women with PCOS, especially for reducing serum androgen levels, a defining characteristic of PCOS [51]. Even if there is a break in practice, this improvement may endure even in the absence of weight reduction. Yoga has been recommended as a therapeutic option for PCOS women due to its effects on hirsutism, abdominal circumference, and hip circumference [52]. By lowering perceived stress and state anxiety and enhancing sleep, yoga treatment significantly affects DUB results [53].

PCOS considerably lessens the feelings of anxiety than a physical exercise regimen [54]. For adult women with PCOS, yoga treatment is useful in maintaining a steady body mass index (BMI) and testosterone level [55]. As a result, Kayakalpa yoga, which involves recycling the seminal return mechanism, is comparable to the pituitary gland's feedback process in synchronizing the hormonal effects of the practice for women with PCOS and infertility [56]. This yogic practice thereby raises and normalizes FSH and LH levels. As a result, when doing Kayakalpa yoga, life force energy is raised [57]. It causes all endocrine glands and the nerve plexus to work, which is beneficial for releasing hormones, maintaining their normal levels, and curing infertility [42]. Regular yoga practice can be utilized as an alternative therapy for promoting health and preventing obesity in teenagers who are overweight [55]. When PCOS patients with autonomic dysfunction practice yoga, their heart rate variability (HRV) significantly improves [56]. Women with PCOS were shown to have significantly different levels of depression before and after receiving yoga practices. There is a significant difference in the level of depression among PCOS-affected women between the pre- and postyoga practicing conditions [17].  Table 1 lists a few examples of yoga techniques, along with the clinical data supporting them that may be useful for PCOS management.

## 5. How Do Herbal Remedies Cure Polycystic Ovary Syndrome?

According to a survey by the PCOS Society, two out of every ten Indian women have PCOS [66]. Six of the 10 women with PCOS who receive a diagnosis are teenagers [67]. According to research from the AIIMS Department of Endocrinology and Metabolism, 20–25% of reproductive-age women had PCOS [68]. Even though 60% of PCOS patients are obese, 35–50% also have fatty livers, 60% to 70% of people have high levels of testosterone, 40% to 60% have glucose intolerance, and 70% have insulin resistance [69]. Although the pathogenesis of PCOS is unclear, some women with the condition have insulin levels that are higher than usual. When insulin levels are too high, the ovaries may create more androgens like testosterone [69]. Women with PCOS frequently battle obesity because of insulin resistance, which might make it more difficult to lose weight [70].

Currently, PCOS is treated with a number of allopathic drugs such as nafarelin, troglitazone, clomiphene, metformin, and spironolactone at the primary level of treatment [71]. However, long-term use of these drugs might result in serious consequences such as menstruation irregularities, nausea, vomiting, gastrointestinal disturbances, weight gain, insulin resistance, and other contraindications [72]. Because of all these complications, people are now moving towards natural treatment including herbal remedies, yoga therapies, lifestyle modifications, and naturopathy which are much more effective than existing allopathic drugs. Herbal treatments have an important role in prevention, treatment, and rehabilitation [73]. However, polyhedral formulations may also be developed on the basis of the aforementioned variables to decrease the cost, duration, and side effects of current treatments.

In general, a plant, its part, or its extract utilized for flavor, fragrance, or medical purposes is referred to as herb in commerce. Traditional herbal remedies are compounds that are naturally occurring and have undergone little to no industrial processing before being utilized to cure a variety of ailments. In addition, herbal treatments are receiving a lot of attention in discussions about global health [1]. Similarly, herbal medications can be a very useful therapeutic option for PCOS also, due to their generally milder effects on the body and fewer side effects than other medicines [74]. It has reached a whirling point and is fighting to be renowned as a science—a particular field with its own uniqueness. It has become imperative to demonstrate how herbal rehabilitation can compete with other medical specialties in terms of the depth of its research and its applicability. The advantage of herbal therapy over normal therapy is that, it is less risky, has fewer complications, and has a potentiating effect due to the existence of many active chemicals in medicinal plants [75].

Herbs may be used for longer periods of time with fewer adverse effects, which is crucial because PCOS requires long-term therapy [76]. They may show promising effects in addressing the causes of PCOS, relieving symptoms, and promoting the body's ability to repair itself by enhancing the immune system. The efficiency of selected herbal medications can be increased by combining them with a PCOS-friendly diet and yoga practises [77]. The use of medicinal plants for both sustenance and the treatment of various ailments is quite significant. Indian and other traditional medicinal systems include several active components from a variety of plant species used to treat PCOS, as mentioned in Table 2.

*Aloe vera*, cinnamon, chamomile, fenugreek, *Heracleum persicum*, Potentilla, *Mentha spicata*, *Foeniculum vulgare*, Licorice, and Marrubium are some of the medicinal herbs that contain active compounds that can affect blood sugar levels, lipid profiles, insulin resistance, hormones, and ovarian tissue afflicted only by *Aloe vera*, chamomile, *Camellia sinensis*, *Mentha spicata*, and silymarin [78].

Evidence-based medical care for polycystic ovary syndrome (PCOS) places a strong focus on a multidisciplinary approach, as standard pharmacological treatment frequently targets a single symptom, may be contraindicated, has adverse effects, and is ineffective in certain circumstances. The old Persian and Chinese medical systems saw the introduction of herbal medicine as a supplemental form of treatment [88]. The treatment of gynecological and reproductive issues in PCOS patients has traditionally included the use of medicinal plants. There were several clinical evidence validated as to the degree to which PCOS and associated symptoms are reduced by Satapushpa Shatavari powder treatment coincides with a commensurate reduction in ovarian volume [89]. In order to control oligomenorrhoea, dry cupping combined with fennel seed infusion is a safe and efficient therapeutic strategy [90]. Cinnamon supplementation improved antioxidant status, serum lipid profile, and menstrual cyclicity in PCOS-affected women, which may be helpful for reducing PCOS risk factors [91]. Green tea use by overweight and obese women with PCOS leads to weight loss, a decrease in fasting insulin, and an increase in free testosterone [92]. Furocyst was effective in reducing PCOS symptoms [93]. In PCOS-afflicted women, cinnamon dramatically decreased fasting insulin and insulin resistance [94]. The natural remedy spearmint may be effective in treating PCOS-related hirsutism [95]. In women with polycystic ovarian syndrome, eating raw red onions seems to be an efficient way to decrease cholesterol [96]. *Nigella sativa* is also a complementary medicine that may help PCOS-affected women with their irregular menstrual cycles [97].  Table 3 provides a summary of several herbs and their clinically proven formulations to cure PCOS.

## 6. How Do Yoga Practices and Herbal Remedies Work Together on Polycystic Ovarian Syndrome Management?

Stress and obesity disrupt the healthy hypothalamic-pituitary-ovarian axis, which causes insulin resistance and a stage of hyperandrogenism that leads to the development of somatic symptoms of PCOS like hirsutism, anovulation, irregular menstruation, subfertility, acne, and psychic symptoms like anxiety, depression, insomnia, and loss of concentration [101]. Losing 5 to 10% of one's body weight can significantly improve PCOS's endocrine profile [102]. Pathophysiological problems, such as hyperandrogenism, insulin resistance, and chronic inflammation in PCOS females, are developed as a result of genetic variation, epigenetic modifications, and a disrupted lifestyle [103]. A single genetic diagnostic technique fails due to the involvement of several proteins, molecules, and signaling pathways at the molecular level in disease development [104]. Four phenotypic variations of PCOS are used to classify PCOS patients into three groups: classic, ovulatory, and nonhyperandrogenic kinds [105]. This genetic method for elucidating the pathophysiology of PCOS was recently discovered. Genetic research can be used to pinpoint the underlying factors that led to the emergence of PCOS.

The primary line of treatment for overweight women with PCOS is lifestyle management, which may control menstruation, lessen hyperandrogenism, treat hyperinsulinemia, and enhance quality of life. With or without dietary and activity improvements, ovulation can return [106]. A decreased prevalence of anovulatory infertility has been associated with regular 30- to 60-minute yoga sessions [107]. Moderate aerobic exercise has a beneficial effect on a variety of cardio-metabolic risk factors in women with PCOS when they do it for more than or comparable to three months [108]. A healthy diet and 30 minutes of moderate-to-vigorous yoga practise performed at least four days a week can help women with PCOS lose weight [109]. However, high attrition in randomized controlled trials (RCTs) limits the robustness of the evidence for lifestyle, and clinical adoption is also hampered by the paucity of data for effective daily yoga and appropriate food therapy [110]. In addition, overweight women—especially those with established obesity—frequently exhibit physical and psychological difficulties. Despite the possible good endocrine consequences of PCOS, many women also employ supplementary therapies, such as herbal treatments. Numerous clinical studies have shown that regular yoga practise helps to reduce stress and obesity, which are the main causes of PCOS [111]. Based on a variety of clinical indications, herbal medicines can also help to restore hormonal balance, considerably reduce inflammation, enhance insulin sensitivity, and promote proper reproductive system function [112].

Yoga is a lifestyle choice that extends much beyond regular yoga practice for the majority of serious practitioners. It creates such a powerful bond between the body, mind, and soul that it frequently permeates all other facets of day-to-day existence [113]. In actuality, the main goal of yoga's development was to link emotional calmness and mental balance to physical wellness. Yoga aims to promote a way of life that places as much emphasis on harmony, gratitude, tranquilly, and positive thinking as it does on physical health. Yoga is highly effective as a unique type of holistic therapy because of this system of priorities [114]. Given that yoga promotes the integration of the mind and body for general wellness, we could even claim that it is a comprehensive integrative healthcare system unto itself. Breath work, meditation, and physical poses are all combined with the entire being in mind [115]. It only makes sense, then, that yoga may be a great alternative therapy to herbal medication. Let us take a look at how these two all-encompassing strategies might be combined for even more advantage.

### 6.1. Herbal Medicine and Yoga Go Hand in Hand

Yoga and herbal treatments appear to be two completely distinct holistic therapies when we examine their historical roots. Yoga did, after all, start in India about 3000 BC. However, it is quite probable that these two methods of thinking were impacted by one another. While each one has own unique cultural roots, they both adhere to the fundamental idea that mental and physical balance is essential for optimum health and wellness. Integrating these two age-old traditions may help practitioners provide more powerful outcomes for their patients [116]. A regular yoga practice that incorporates herbal treatments might assist you in developing a deeper awareness of your body.

If a person has been practicing yoga for some time, they presumably already recognize the link. Yoga's core tenet is the promotion of both physical and mental well-being via the appropriate movement of energy throughout the body. Restful postures are utilized in yoga and are frequently focused on the body's meridians while being maintained for many minutes [117]. Additionally, some emotions like fear, rage, and enthusiasm are connected to herbal remedies. Excessive emotions are supposed to throw off the body's equilibrium. To regulate the autonomic nervous system and encourage energy flow, herbal medicine practitioners and practitioners of traditional medicine treat the organ and the emotions related to the organ [110]. In yoga, we continue to utilise both the body and the mind to encourage harmony and balance.

### 6.2. How Can Integrated-Pathy Be Useful in the Management of PCOS?

Meditation is a crucial strategy for achieving emotional balance and mental quiet that is used in both yoga and herbal medicines [118]. As a result, when yoga and herbal remedies are used together, they can be quite effective in managing PCOS. There are several clinical studies which may support the use of herbal medicine in combination with a healthy lifestyle to treat PCOS. Connecting the mind and body is the main focus of both disciplines.

Modern culture frequently thinks that conventional medicine is superior to holistic modalities like yoga and herbal medicines. However, these integrative and holistic approaches can improve PCOS more profoundly and for a longer period of time. Changes in behavior and lifestyle, in particular, can significantly enhance the quality of life for those with chronic diseases. The symptoms of this condition can be slowed down or even reversed by making lifestyle changes, such as practicing yoga and using conventional herbal medicines. Additionally, holistic treatments might be particularly helpful when mainstream medicine is unable to identify a problem. This is so that systemic imbalances, both mentally and physically, may be addressed through these therapies. In our culture, taking a pill is a normal approach for managing PCOS, although this typically only offers transient relief. Combining these comprehensive strategies may result in a more effective strategy for long-lasting improvement and enhanced mental and physical wellness.

**Figure 8:**
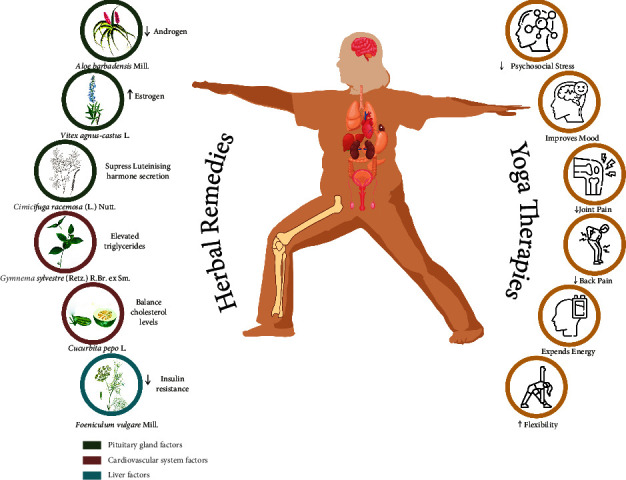
Combinational mechanism of herbs and yoga practices for PCOS management.

As this review discussed, women with PCOS require individualised treatment employing yoga practices and herbal remedies. There are several number of clinical evidence which supports yoga practices and herbal remedies individually effective in the PCOS management. By mixing yoga and herbal treatments into daily life, as briefly shown in Figure 8, it might be possible to treat PCOS and other female health disorders safely and effectively. Hence, the integrated-pathy of yoga and herbs in PCOS may offer significant advantages to women.

## 7. Adverse Effects of Allopathic Drugs Used in Polycystic Ovarian Syndrome

The negative consequences of modern medicine on the treatment of PCOS have been reported in a number of cases. A few examples of such cases include liver toxicity with Flutamide, and a higher relative risk difference for venous thromboembolism with oral contraceptives. In a case study, one hirsute adolescent with significant hepatotoxicity induced by Flutamide demonstrated a relationship between the length of the medication and liver damage [119]. In another case, a 14-year-old girl had a dermatological consultation and was given flutamide (500 mg, orally) for the treatment of facial hirsutism which leads to fulminant hepatic failure [120]. In another clinical evidence, 626 infertile women with PCOS received metformin and clomiphene alone or in combination up to 6 months. Women were withdrawn from the trial with some adverse effects due to the metformin and Clomiphene. The side effects of clomiphene on the gastrointestinal tract include abdominal pain and discomfort in 53% of women, diarrhoea (23%), nausea (39%), hot flushes (28%), headaches (44%), flatulence (18%), and mood swings (15%). For metformin, side effects include abdominal pain and discomfort (59%), diarrhoea (65%), nausea (62%), vomiting (30%), flatulence (18%), headaches (42%), and mood swings (17%). As a result of serious adverse event due to the administration of metformin includes one death [121].

## 8. Conclusion and Future Perspectives

Polycystic ovary syndrome (PCOS) is the most frequent endocrinopathy in women, and most people are unaware that it has become a worldwide epidemic. Women with PCOS often do not receive proper treatment, as few understand its complexity. Conventional medical therapies are limited in quantity and efficacy, with significant associated risks. Prevention is the ultimate solution, requiring drastic changes and the elimination of endocrine disruptors and ever-present toxic foods. Minor lifestyle changes along with herbal treatment and yoga therapies can improve the symptoms in PCOS patients. Yoga and lifestyle modification should be considered for first line interventions for PCOS with or without medical interventions, especially in young patients. By adopting a holistic approach that addresses both lifestyle factors and herbal support, women with PCOS can take proactive steps towards hormonal balance, symptom relief, and overall well-being with no side effects and at a cost that is cost-effective. The integrated approach of lifestyle modification and herbal remedies may hold promise for managing PCOS and improving the quality of life for women affected by this condition. In order to determine the effectiveness of herbal remedies combined with yoga practices for PCOS, more preclinical and clinical research is required. Clinical trials and long-term studies can provide more robust evidence regarding the specific benefits of different herbs and their mechanisms of action.

## Figures and Tables

**Table 1:** Clinical evidence of different yoga therapies involved in PCOS management.

S. Nos.	Study design	Yoga therapy	Duration of therapy	No. of patients involved	Interpretation	Authors/study year
1	Randomized, interventional controlled	Suryanamaskara, asanas, pranayama, meditation	1 hr./day (12 weeks)	90 adolescent females (15–18 years)	↑ Insulin resistance, lipids, glucose, and other measurements in teenage PCOS females without regard to anthropometric alterations	Nidhi et al. 2012 [17]

2	Randomized active controlled	Pranayama, relaxation	1 hr./day (12 weeks)	90 adolescent females (15–18 years)	↓ LH, AMH, testosterone, mFG score for hirsutism; ↑ Menstruation frequency in teenage PCOS with no changes in FSH, body weight, and prolactin	Nidhi et al. 2013 [50]
	Randomized controlled		1 hr., thrice weekly (3 months)	21 women (23–43 years)	↑ Diet and serum androgen levels	Patel et al., 2020 [51]
	Randomized, active interventional controlled		1 hr./day (12 weeks)	90 adolescent females (15–18 years)	↓ Anxiety symptoms	Nidhi et al. 2012 [58]

3	Randomized controlled	Makarasana, bhujangasana, dharmikasana, paschimottanasana, sardulasana, bhadrasana, matsyedrasana, and savasana	45 min. (6 weeks)	61 women (mean age 30.77 ± 6.01 years)	↑ Hirsutism, abdominal, and hip circumference	Mohseni et al. 2021 [52]

4	Parallel-arm controlled pilot	Shithalikaran vyayama, suryanamaskara, ardhakati chakrasana, badhakonasana, bhujangasana, viparita karani, sarvangasana, surya anulomsa viloma, kapalabhati, and meditation	3 consecutive months for 60 min. a day for 3 days a week	30 women (20–40 years)	↓ Perceived stress and anxiety levels	Nalgirkar et al. 2018 [53]

5	Interventional	Vamana karma	2 months	15 women (20–40 years)	↓ FBS level; Body weight and body mass index (BMI) and regularising menstruation	Bhingardive et al. 2017 [59]

6	Pilot	Pranayama, meditations, bhadrasana, and chakki chalanasana	2 months	102 adolescent females	↓ Risk of PCOS through yoga and fitness regimens	Selvaraj et al. 2020 [60]

7	Covariance	Surya namaskar, asana, pranayama and yoga nidra	60 min., six days a week (8 weeks)	30 women (20–35 years)	↓ Body mass index and testosterone levels in adult PCOS women	Shalini and Elangovan 2020 [61]

8	Pilot	Kayakalpa	3 months	10 women (20–30 years)	↓ Body weight, maintains proper FSH and LH balance, regulates aberrant cholesterol levels, and controls regular menstruation	Shanthi and Perumal 2014 [56]

9	Single-center, single-blinded randomized controlled	Asanas, pranayama, and surya namaskar yoga	3 times per week (12 weeks)	40 adolescent females (19–22 years)	↑ Muscular mass; ↓ Body fat percentage and body mass index	Na Nongkhai et al. 2021 [62]

10	Prospective intervention pilot	AgniSar and kapalbhati kriya, shavasana, .pranayama, suryabedhan, anulomvilan, bhramari, om chanting and maun	60 min. on 6 days a week (12 weeks)	30 women (20–35 years)	↑ Cardiac autonomic function with parasympathetic dominance	Verma et al., 2019 [63]

11	Case	Salabhasanam and pranayama	30 minutes twice a day for 1 year	25-year-old female	↓ Face acne, body weight and menstrual cycle interval	Thilagavathi 2019 [64]

12	Randomized, active interventional controlled	Bhadrasana, bharadvajasana, bhujangasana, naukasana, padmasana, dhanurasana, viparitaShalabhasana, chakki chalanasana, sun salutation, shavasana, bhramri pranayama, and meditation	1 month	30 women (25–35 years)	↓ Level of depression	Sode and Bhardwaj 2017 [65]

**Table 2:** Summary of some common herbs beneficial to curing polycystic ovary syndrome.

S. Nos.	Plant families	Plant species	Common names	Parts used	Active constituents	Mechanism of action	References
1	Leguminosae	*Glycyrrhiza glabra* L.	Liquorice, mulhatti	Roots	Glycyrrhizin, glycyrrhizic acid	↓ Blood hormone levels by impairing 11-hydroxysteroids dehydrogenase; Boosting aromatase or progesterone-like actions	[78]

2	Liliaceae	*Aloe barbadensis* Mill.	Aloe vera	Leaves	Phytosterols	Modify steroidogenic response; Express estrogen receptor protein; ↓ Androgens; ↑ Estrogen	[78]

3	Linaceae	*Linum usitatissimum* L.	Linseed	Seed	Lignan	↓ Insulin, total and free serum testosterone levels, and BMI (body mass index)	[79]

4	Apocynaceae	*Gymnema sylvestre* (Retz.) R.Br. ex Sm.	Gymnema	Leaves	Gymnemic acid	↓ High triglyceride levels linked to PCOS	[80]

5	Apiaceae	*Foeniculum vulgare* Mill.	Fennel, shatapushpa	Seeds	Palmitic acid, phytoestrogen	↓ Testosterone levels; Prevent the development of dihydrotestosterone receptor complex; ↓ Insulin resistance and PCO inflammation aids in lessening the cellular imbalance that causes PCOS metabolic abnormalities	[78]

6	Lauraceae	*Cinnamomum zeylanicum* Blume	Cinnamon	Bark	Procyanidine polyphenols	↑ Glycogen synthesis and hypoglycemic effect	[81]

7	Lamiaceae	*Vitex agnus-castus* L.	Chaste berry	Fruits	Flavonoids, carotenoids	Promote and maintain pituitary gland's proper operation, which releases luteinizing hormone	[82]

8	Urticaceae	*Urtica dioica* L.	Stinging nettle	Aerial parts	Flavonoids, tannins, sterols	↑ Formation of SHBG (sex hormone-binding globulin), which lowers the blood level of free testosterone and aids in the normalization of hormone levels	[74]

9	Fabaceae	*Trifolium pratense* L.	Red clover	Aerial parts	Isoflavones	Purification of blood and acne management	[74]

10	Ranunculaceae	*Cimicifuga racemosa* (L.) Nutt.	Black cohosh	Root	Flavonoids and triterpenes as 27-deoxyactein	Suppress luteinizing hormone secretion	[83]

11	Theaceae	*Camellia sinensis* (L.) Kuntze	Green tea	Leaves	Catechins	↓ Hormone levels that lead to ovarian cysts and other symptoms	[78]

12	Phyllanthaceae	*Phyllanthus emblica* L.	Amla	Fruit	Polyphenols	Anti-inflammatory and free radical scavenging effects that support body's restoration of hormonal homeostasis	[74]

13	Pedaliaceae	*Sesamum indicum* L.	Sesame	Seeds	Polyunsaturated fatty acids	Regulation of blood glucose levels	[74]

14	Cucurbitaceae	*Cucurbita pepo* L.	Pumpkin	Seeds	Omega-3 fatty acids	Control the elevated insulin and lipid levels	[84]

15	Lamiaceae	*Ocimum tenuiflorum* L.	Tulsi	Leaves, stems	Eugenol	Moderate insulin levels and manage androgens	[74]

16	Leguminosae	*Trigonella foenum-graecum* L.	Fenugreek	Seeds	Soluble fibers	↓ Blood sugar by limiting enzymatic breakdown and absorption of carbohydrates	[78]

17	Asteraceae	*Silybum marianum* (L.) Gaertn.	Blessed thistle or milk thistle	Seeds	Silymarin	Influences glucose 6-phosphatase; ↓ Gluconeogenesis, blood glucose, and other PCOS symptoms	[78]

18	Asteraceae	*Matricaria chamomilla* L.	Chamomile	Flowers	Sterols, ascorbic acid	Stop gaining weight; ↓ Cholesterol level	[85]

19	Apiaceae	*Heracleum persicum* Desf. ex Fisch., C.A.Mey. & Avé-Lall.	Persian hogweed or golpar	Roots	Furanocoumarins as sphondin, xanthotoxin, pimpinellin	↓ Production of nitric oxide (NO), LH level, and estradiol release	[78]

20	Lamiaceae	*Mentha piperita* L.	Peppermint	Flowering aerial part	Essential oils	↑ Cytochrome P450 3A4 (CYP3A4) activity, which changes the concentration of androgen and steroid hormones; ↓ Free testosterone levels; ↑ Sex hormone-binding globulin	[78]

21	Rosaceae	*Potentilla fruticosa* L.	Shrubby cinquefoil	Fruits/seeds	Vitex, lactone	Prevents prolactin from being released and lessens fibrocystic mastopathy by interacting with dopamine receptor D2 (D2 R) in the hypothalamus and glandular pituitary	[78]

22	Lamiaceae	*Marrubium vulgare* L.	White horehound	Leaves	Apigenin, ursolic acid	Inhibit cytochrome P450 enzyme, which prevents cholesterol from being converted to pregnenolone; ↓ Synthesis of steroid hormones like progesterone	[78]

23	Asparagaceae	*Asparagus racemosus* Willd.	Satavari, satawar	Roots	Phytoestrogen	Encourages healthy ovarian follicle formation, controls menstrual cycle, and reenergizes the female reproductive system	[86]

24	Menispermaceae	*Tinospora cordifolia* (Willd.) Hook.f. & Thomson	Guduchi	Leaves	Berberine, rumphioside I, syringin	↑ Metabolism; ↓ Insulin resistance and renewing all body tissues	[86]

25	Ranunculanae	*Actatea racemosa* L.	Black cohosh	Rhizomes, roots	Triterpenoids, isoflavones, aglycones	Encourage ovulation in PCO-afflicted women	[86]

26	Brassicaceae	*Lepidium meyenii* Walp.	Maca	Rhizome	Imidazole alkaloids, macamides, macaenes	Menopause symptoms are relieved, the endocrine system is stimulated, and without causing any negative effects, it naturally balances hormones	[86]

27	Asteraceae	*Taraxacum officinale* F.H.Wigg.	Dandelion	Roots	Vitamins A, C, and K	↑ SHGB synthesis to lower blood levels of free testosterone. Removal of toxins from the body	[86]

28	Asclepiadaceae	*Pergularia daemia* (Forssk.) Chiov.	Veli paruthi	Leaves, stems, shoots, roots, seeds, fruits	Terpenoid, flavonoids, sterols, cardenolids	↓ Testosterone and LH levels; ↑ Progesterone and FSH levels	[86]

29	Arecaceae	*Areca catechu* L.	Betal palm	Fruits	Arecoline	Sustain a strong libido and menopausal transition; aids in uterine retentive power; treat postpartum debility	[86]

30	Ranunculaceae	*Nigella sativa* L.	Kalonji	Seeds	Thymoquinone, thymol, unsaturated fatty acids, lipase and tannins	Beneficial for insulin resistance syndrome; helps to reduce fat	[87]

31	Lamiaceae	*Mentha spicata* L.	Pudina, spearmint	Leaves	Carvone, limonene	↓ Free and total testosterone levels, hirsutism intensity	[78]

**Table 3:** Clinical evidence of different herbs involved in PCOS management.

S. nos.	Study designs	Name of species	Formulations/parts used	Doses/route of administration	No. of patients involved	Interpretation	Authors/study year
1	Randomized single blind	*Anethum graveolens* L., *Asparagus racemosus* Willd.	Powder (seeds, roots)	5 g t. d. s. with 10 ml of cow's ghee for (2 weeks)	60 patients (18–42 yrs.)	↓ Ovarian volume	Kumarapeli et al. 2018 [89]

2	Randomized controlled	*Foeniculum vulgare* Mill.	Infusion (seeds)	5 g with 200 ml boiling water/once a day	61 patients (mean age: 26.68)	↓ Menstrual cycle duration in women with oligomenorrhoea	Mokaberinejad et al. 2019 [90]

3	Randomized controlled	*Foeniculum vulgare* Mill., *Urtica dioica* L., *Daucus carota* L.	Sachet	5 g/day (12 weeks)	80 patients (15–40 yrs.)	↓ Insulin resistance assessed by homeostasis model; ↓ Fasting insulin, total cholesterol, low-density lipoprotein cholesterol, body fat, and body mass index; ↓ Levels of triglycerides, alanine aminotransferase, aspartate aminotransferase	Rouhani et al. 2019 [98]

4	Double-blind randomized controlled	*Cinnamomum zeylanicum* Blume	Capsule (bark)	One capsule 3 times a day (8 weeks)	84 women (20–38 yrs.)	↓ Malondialdehyde; ↑ Total, low-density, high-density lipoprotein cholesterol levels in the blood	Borzoei et al. 2018 [91]

5	Double-blind, randomized	*Camellia sinensis* (L.) Kuntze	Tablets	500 mg green tea capsule	60 women (20–40 yrs.)	↑ Free testosterone level; ↓ Fasting insulin	Allahdadian et al. 2017 [92]

6	Open label, one-arm, nonrandomized, postmarketing surveillance	*Trigonella foenum-graecum* L.	Capsules (seeds)	2 capsules of 500 mg each/day	50 women (18–45 yrs.)	↓ Ovarian volume and ovarian cyst count	Swaroop et al. 2015 [93]

7	Randomized double-blind placebo-controlled	*Cinnamomum cassia* (L.) J.Presl	Capsules (bark)	1.5 g/day in 3 divided doses (12 weeks)	66 women	↓ Insulin and LDL levels; ↑ Insulin sensitivity	Hajimonfarednejad et al. 2018 [99]

8	Randomized controlled	*Mentha spicata* L.	Spearmint tea (Leaves)	Twice a day (1 month)	42 women (19–42 yrs.)	↓ Testosterone levels and hirsutism scores on the modified DQLI	Grant 2010 [95]

9	Randomized controlled	*Allium cepa* L.	Raw red onions	8 weeks	54 women (17–37 yrs.)	↓ Total cholesterol levels	Ebrahimi‐Mamaghani et al. 2014 [96]

10	Placebo-controlled, double-blinded randomized	*Cinnamomum verum* J.Presl	Cinnamon supplements (inner bark)	1.5 g/day (6 months)	45 women	Improves menstrual cycle	Kort and Lobo 2014 [100]

11	Double-blinded placebo-controlled	*Nigella sativa* L.	Capsules	500 mg (16 weeks)	32 women (18–38 yrs.)	Menstrual cycles occurred substantially more frequently in the intervention group (0.79) than in the control group (0.48)	Naeimi et al. 2020 [97]

## Data Availability

The data supporting this review are from previously reported studies and datasets, which have been cited.
